# Microencapsulation of *Lactococcus lactis* Gh1 with Gum Arabic and *Synsepalum dulcificum* via Spray Drying for Potential Inclusion in Functional Yogurt

**DOI:** 10.3390/molecules24071422

**Published:** 2019-04-11

**Authors:** Nurul Farhana Fazilah, Nurmelissa Hanani Hamidon, Arbakariya B. Ariff, Mohd Ezuan Khayat, Helmi Wasoh, Murni Halim

**Affiliations:** 1 Department of Bioprocess Technology, Faculty of Biotechnology and Biomolecular Sciences, Universiti Putra Malaysia, Selangor 43400, Malaysia; farhanafazilah93@gmail.com (N.F.F.); nmelissahanani@gmail.com (N.H.H.); arbarif@upm.edu.my (A.B.A.); helmi_wmi@upm.edu.my (H.W.); 2 Bioprocessing and Biomanufacturing Research Center, Faculty of Biotechnology and Biomolecular Sciences, Universiti Putra Malaysia, Selangor 43400, Malaysia; m_ezuan@upm.edu.my; 3 Department of Biochemistry, Faculty of Biotechnology and Biomolecular Sciences, Universiti Putra Malaysia, Selangor 43400, Malaysia

**Keywords:** microencapsulation, spray drying, probiotic, gum Arabic, *Synsepalum dulcificum*, functional food, yogurt, *Lactococcus lactis*, *Lactobacillus delbrueckii* subs. *bulgaricus*, *Streptococcus thermophilus*

## Abstract

There has been an explosion of probiotic incorporated based product. However, many reports indicated that most of the probiotics have failed to survive in high quantity, which has limited their effectiveness in most functional foods. Thus, to overcome this problem, microencapsulation is considered to be a promising process. In this study, *Lactococcus lactis* Gh1 was encapsulated via spray-drying with gum Arabic together with *Synsepalum dulcificum* or commonly known as miracle fruit. It was observed that after spray-drying, high viability (~10^9^ CFU/mL) powders containing *L. lactis* in combination with *S. dulcificum* were developed, which was then formulated into yogurt. The tolerance of encapsulated bacterial cells in simulated gastric juice at pH 1.5 was tested in an in-vitro model and the result showed that after 2 h, cell viability remained high at 1.11 × 10^6^ CFU/mL. Incubation of encapsulated cells in the presence of 0.6% (*w*/*v*) bile salts showed it was able to survive (~10^4^ CFU/mL) after 2 h. Microencapsulated *L. lactis* retained a higher viability, at ~10^7^ CFU/mL, when incorporated into yogurt compared to non-microencapsulated cells ~10^5^ CFU/mL. The fortification of microencapsulated and non-microencapsulated *L. lactis* in yogurts influenced the viable cell counts of yogurt starter cultures, *Lactobacillus delbrueckii* subs. *bulgaricus* and *Streptococcus thermophilus*.

## 1. Introduction

Recently, probiotic-based food has gained a lot of popularity among consumers worldwide. Probiotics is defined as, “live microorganisms that, when administered in adequate amounts, confer a health benefit on the host’’ [1]. Probiotic has been incorporated into many types of foods, including dairy products, such as yogurt, and non-dairy products, such as chocolate [2]. Lactic acid bacteria (LAB) are among the most significant groups of probiotic organisms and *Lactobacillus*, *Bifidobacterium*, *Pediococcus*, *Lactococcus*, and several other genera have been proposed and used as probiotic strains [3,4].

Consumption of probiotic via food products is a good way to re-establish the intestinal microflora. Nonetheless, the main challenges of developing any probiotic food products are to improve the viability of culture during processing, storage, and until it reaches the gastrointestinal tract of humans, as well as finding a cost-effective way in producing these foods [2,5]. Probiotic cells must be protected from adverse environments, and to avoid any negative sensory impact when incorporated into foods [6]. These criteria can be met by applying encapsulation technology, such as spray drying techniques. The spray dryer system has been presented as an efficient method for encapsulation in the food industry. Spray drying provides rapid evaporation of water and maintains low temperature in the particles [7]. It involves atomization of feed solution into the hot air drying chamber, wherein evaporation of water takes place from the atomized droplets to form dry powder [8]. Spray drying offers many advantages, as it is easy for scale up, production of flowable powders, the ability to control particle size, rapid drying, and continuous production. Most importantly, the dried encapsulated probiotic bacteria can reduce storage and transportation cost [9]. There are numerous types of wall material that can be used for encapsulating agents in the spray drying process. An ideal encapsulant should possesses emulsifying and film-forming properties, biodegradable, resistant to gastrointestinal tract, low viscosity at high solid contents, exhibit low hygroscopicity, and most importantly, it must also be low cost [10]. According to Tonon et al. [11], the most commonly used encapsulants for juices and fruit extracts are maltodextrins and gum Arabic. Gum Arabic is mainly used because of its high water solubility characteristics, low viscosity, and emulsifying properties [12].

*Synsepalum dulcificum*, commonly known as miracle fruit, is unique as it can convert a sour taste to a sweet taste. Glycoprotein, known as miraculin, which is found in the pulp of this fruit is the compound responsible for this unique taste modifying function [13,14,15]. The binding of miraculin to the receptor cells of the tongue suppresses the response of a sour taste in the central nervous system. The effect would last until the miraculin is diluted and eliminated by saliva. The taste modification function provides a great potential for this fruit to be exploited in the food industry. The locals in West Africa have traditionally used the fruit to sweeten sour foods and beverages [16]. Miracle fruit’s pulp and seed contain high nutrient contents, which can be used for dietary supplements [17]. The pulp of miracle fruit has been reported to have a large amount of vitamin C (40.1 mg/100 g FW), close to that in citrus fruits (33–43 mg/100 g FW) [15]. Compared with other fruits that are known for their rich source of antioxidant phenolic, such as berries (i.e., blackberry (435 mg GAE/100 g FW) and bluberry (348 mg GAE/100 g FW), the total phenolic in the skin and pulp of miracle fruit is much higher with 625.57 mg GAE/100 g FW. The total flavonoid contents of miracle fruit pulp (9.9 mg of QR Equiv/100 g FW) and seed (3.8 mg of QR Equiv/100 g FW) are also considerably high [13]. The stem of miracle fruit also contains antioxidant and an antityrosinase effect, which can have a potential application in food supplements and medical cosmetology [18]. Besides antioxidants, the polysaccharides from the miracle fruit leaf have been characterized to have an α-glucosidase inhibitory activity that was remarkably higher than acarbose (antidiabetic drug), which has highlighted its potential as an anti-diabetic agent [19]. The low levels of crude fat [20] and sugar content (5.6 g/100 g FW) [15] in the pulp of miracle fruit indicate that it may be healthy for human consumption, especially for patients suffering from diabetes and obesity. Chen et al., [21] reported that an animal model study on the improvement of insulin resistance in fructose-rich chow-fed rats by the consumption of miracle fruit. Three-time daily oral administration of 0.2 mg/kg miracle fruit has proven the ability of this fruit to improve insulin sensitivity more rapidly than that of metformin (oral hypoglycemic agent for treating diabetic patients). In the meantime, the high water content in the fresh miracle fruit pulp (in the range of 60–70%) [15,20,22] implies that this fruit may have a short shelf-life due to its moisture content. Hence, for commercial applications, the dehydration process may be applied to improve the shelf life. Furthermore, dehydration process may also increase the level of crude fiber content (12.5 g/100 g fresh fruit weight) of the fruit [15], which may enhance fecal bulk and the rate of intestinal transit. It can also provide a prebiotic effect upon consumption or when formulated into food products. Nevertheless, further research is needed to discover the ability of miracle fruit as a prebiotic agent. Miracle fruit parts may hence be mixed with encapsulating agents during the encapsulation process and formulated into functional food, such as yogurt, to improve its nutritional and therapeutic properties.

Yogurt is a widely consumed functional food, due to its good taste and nutritional properties and has beneficial effects on human health. Yogurt eating has been associated with several health benefits including improvement of lactose metabolism, anti-mutagenic properties, anti-carcinogenic properties, management of hypertension, anti-diarrheal properties, immune system stimulation, and improvement in inflammatory bowel disease [23,24]. The beneficial health effects of yogurt have been partly linked to the proteolysis products produced during fermentation and storage [2]. Modern yogurt fully utilizes ingredients, such as milk, milk powder, sugar, fruit, flavors, coloring, emulsifiers, stabilizers, and specific pure starter cultures of LAB in a well-controlled process of fermentation [25]. The conventional yogurt starter culture strains (i.e., *Streptococcus thermophilus* and *Lactobacillus delbrueckii* subs. *bulgaricus*) lack the ability to pass through the intestinal tract [26] and hence may not play a significant role as probiotics in the human gut, as they are incapable of colonizing the human intestine [27]. Therefore, the current trend is to add other probiotic strains during yogurt fermentation along with the starter culture bacteria to induce the probiotic effect.

The purpose of this study was hence to investigate the characteristics of microencapsulated *Lactococcus lactis* Gh1 in the presence of gum Arabic and miracle fruit plant parts via spray drying technique. *L. lactis* Gh1 is a potential probiotic strain isolated from ghara, a traditional flavor enhancer prepared from whey [28]. The strain was determined to be able to hydrolyze mannitol, sucrose, ribose, d-xylose, galactose, mannose, glucose, fructose, trehalose, lactose, maltose, cellobiose, gentiobiose, gluconate, n-acetyl-glucosamine, amygdalin, esculin, arbutin, salicin and starch. *L. lactis* Gh1 also showed an inhibitory effect against a food-borne pathogen, *Listeria monocytogenes*. Furthermore, *L. lactis* Gh1 has the ability to coagulate milk and was observed to be susceptible to a wide range of antibiotics, haemolytic, amylolytic, and proteolytic activities and was tolerant to NaCl (up to 4.0% (*w*/*v*)), bile salt, phenol and low pH conditions [29]. The spray dried *L. lactis* Gh1, in conjunction with the added miracle fruit plant parts and gum Arabic as encapsulating agents, were then formulated as a functional yogurt.

## 2. Results and Discussion

### 2.1. Survivability of Encapsulated L. lactis Gh1 After Spray Drying

Suitable wall materials are crucial in protecting microbial cells from denaturing, due to the high temperatures during the spray drying process. Gum Arabic was particularly chosen as an encapsulanting agent, as the spray-dried gum Arabic encapsulated lactic acid bacteria (LAB) and was previously reported to result in the highest survivability percentage compared to skim milk, gelatin, and soluble starch [30]. In the mean-time, miracle fruit plant part extracts were supplemented to assess their influences on the gum Arabic as the major structural ingredient in the encapsulating agent used for *L. lactis* Gh1. As reported in our previous studies, the fruit has been characterized with high nutritional elements [15] and healthy sweet-inducing activity from the presence of miraculin compound [14], that may provide health benefits when incorporated into food product. During the spray drying process, a low percentage of gum Arabic solution is often used to avoid clogging of the sprayer [31]. Spray dried *L. lactis* Gh1, encapsulated with gum Arabic and miracle fruit seed (MFS), showed the highest survival of *L. lactis* (85.00%), followed by spray dried *L. lactis*, encapsulated with gum Arabic and miracle fruit pulp (MFP) (36.36%), and spray dried *L. lactis* encapsulated with gum Arabic and miracle fruit leaf (MFL) (36.04%) (Table 1). The lowest cells survival was observed for spray dried *L. lactis* encapsulated with gum Arabic (GA) only. These corresponded to an encapsulation efficiency of 99.27%, 95.44%, 95.43%, and 94.05%, respectively. The presence of miracle fruit parts (seed, leaf, and pulp) have enhanced the survivability percentage compared to the spray dried solution containing only gum Arabic. Among the three parts of miracle fruit, the seed had showed the highest survivability of approximately ~49% in contrast with leaf and pulp. This result may be related to the oil content that is present in the seed, as reported by He et al., [15]. The miracle fruit seed oil composed of oleic acid, palmitic acid, and linoleic acid as the major fatty acid components and have a highly similar triacylglycerol (TAG) profile to palm oil. It was reported that the addition of lipid to carbohydrate and/or protein, as a mixture of encapsulating agent during drying, produced a more stable microcapsule compared to the matrix formulated without lipid [32]. For example, the addition of coconut oil to gum Arabic and gelatin mixture helped to improve the viability of probiotic cells during spray drying process [33]. Overall, it was noted that the reduction of viable cells after spray drying process was slight, all less than 1.0 log CFU/mL. This demonstrates that the cells did not suffer much damage from the heat applied during the spray drying process in which the outlet and inlet temperatures were set at 130 °C, and 60 °C, respectively. Even though the survival percentage was lower for GA, MFL, and MFP, they still attained higher viable cells than the minimum level of viable probiotic cells (in between 10^6^ to 10^7^ CFU/mL) requirement for food incorporation to be claimed as probiotic product [2].

### 2.2. Morphology

**Figure 1:**
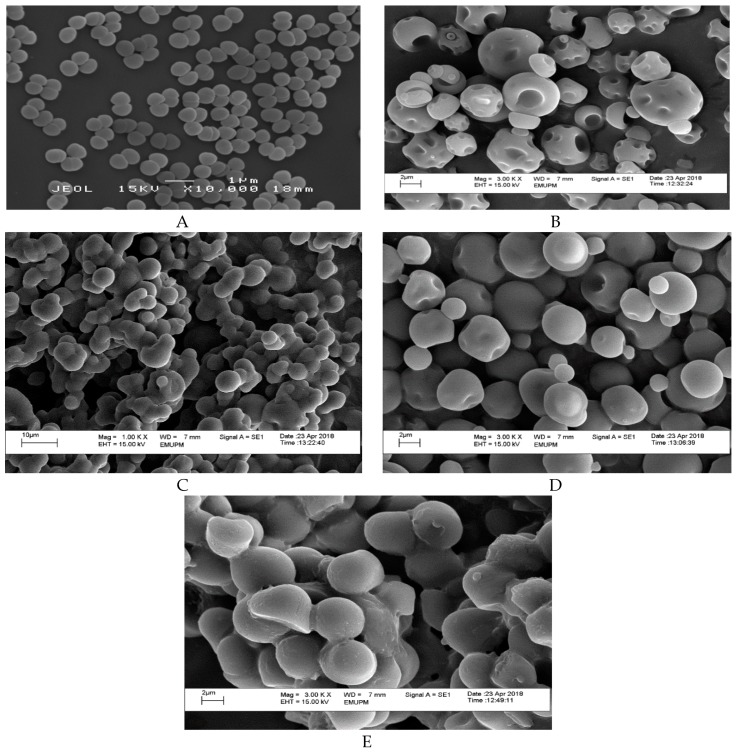
Scanning electron micrographs (SEM) of (**A**) non-encapsulated *L. lactis* Gh 1 cells (free cell); and spray dried encapsulated *L. lactis* Gh 1 with (**B**) 5% gum Arabic; (**C**) 5% gum Arabic and 5% miracle fruit pulp; (**D**) 5% gum Arabic and 5% miracle fruit leaf; (**E**) 5% gum Arabic and 5% miracle fruit seed. Encapsulated and non-encapsulated cells (free cell) were magnified at 3,000×, and 10,000×, respectively.

Morphology structure affects the flow ability, particle size, and particle friability characteristics of encapsulated cell powder [34]. Figure 1 depicted the morphology of the encapsulated and non-encapsulated (free cell) *L. lactis* Gh1, formed after spray drying technique. A smooth surface was observed in *L. lactis* cell powder, encapsulated with either miracle fruit pulp, leaf, or seed in the presence of gum Arabic (Figure 1C–E), whereas a dent surface can be clearly observed on the GA *L. lactis* cell particle (Figure 1B). Similar results on the dent surfaces of gum Arabic were previously reported by Rascón et al. [35], Ferrari et al. [36], and Bhusari et al. [37]. The dent was formed due to a high water evaporation rate in spray drying, followed with the shrinkage of particles [38]. The combination of different wall materials might contribute to the surface formation of spray dried powder. This finding is in complete agreement with Kuck and Noreña [38] where they observed the combination of polydextrose and guar gum had produced more spherical particles with few dents and roughness.

The intact spherical shape indicates that the cells were fully protected from the external environment and hence gas permeability is expected to be lower. Moreover, from Figure 1C–E, the encapsulated cell showed spherical shapes on the whole and the particles had a complete surface structure with no cracks or collapses, which suggested that the microencapsulation of powder may be favorable and effective. According to Fernandes et al. [39], microcapsules, that have a wrinkled surface after the spray drying process, are the reason for poor fluidity of the microencapsules. Thus, from this result, a good fluidity of microencapsules was expected in *L. lactis* cell powder contained miracle fruit pulp.

### 2.3. Moisture Content of Encapsulated L. lactis Gh1

Moisture content is an essential parameter to determine the dried powder stability, as it influences the chemical and mechanical aspects [40]. Excess moisture produces sticky powder products that often result in clumping once the dried powder is exposed to the environment and hasten the degradation of the product. From the result obtained, sample GA showed the highest moisture content compared with the other samples (Table 2). In this study, a constant feed rate of spray drying was used with the inlet temperature set at 130 °C. A study on spray drying of pineapple juice by Jittanit et al., [41] observed that moisture content was reduced when the feed flow was kept at a constant speed with increasing inlet temperature. This observation is in agreement with the results obtained, as gum Arabic mixed with miracle fruit parts, exhibited lower moisture content compared with the solution containing only gum Arabic. Furthermore, the higher the inlet temperature, the higher the temperature between the feed and drying air, which increases water evaporation thus producing powder with a lower moisture content [11].

### 2.4. Water Activity

The determination of water activity (*a_w_*) in food powders is very important as this parameter influences their stability in the presence of microbial and chemical ingredients. Both chemical and microbial ingredients are linked to the quality of the dried products, as the rate of its decrease begins above 0.3 for some chemical reaction [42]. In the current study, the *a_w_* of the entire samples were below 0.3, indicating the possible inhibition of microbial growth in the samples obtained. As summarized in Table 3, the highest water activity can be observed from the GA sample. The supplementation of miracle fruit leaf and seed to gum Arabic as encapsulants during spray drying, significantly helped to further reduced the *a_w_* of powders produced. The number of viable cells in MFS (3.4 × 10^9^ CFU/mL) was the highest compared to the other samples. This result is in an agreement with Abe et al. [43], that stated the survival rate of cells is higher at lower *a_w_*.

Water activity concept is considered as one of the most important parameters in food preservation of dehydrated products [44]. In general, dry food has a low *a_w_* in the range of 0.2. For probiotics, the *a_w_* recommended for a long term storage should be around 0.25 [45]. The results obtained in this study almost meet this requirement. On the other hand, Ananta et al. [46], stated that moisture content and a_w_ should be lower than 5%, and 0.3, respectively to avoid agglomeration problems during storage and transportation. The values obtained from this study are also between the suggested values. It is predicted that the *a_w_* of the powder samples may be lowered by increasing the percentage of miracle fruit plant parts as encapsulants. Hence an optimization study on the percentage of encapsulants used during the spray drying process should be conducted to confirm this.

### 2.5. Hygroscopicity

Hygroscopicity measures the adsorption of moisture in the atmosphere and is often expressed as g of water adsorbed per 100 g of dry solid (g/100 g). From the results obtained in this study, there was no obvious difference observed. However, it can be seen that the addition of miracle fruit plant parts increased the moisture gain. This may be due to phenolics content in the plant parts. Chang et al. [47], studied mulberry leaves and *Lucid Ganoderma* and suggested that hygroscopicity might be related to the content of phenolics and their glycosides components. When comparing the data in Table 2 and Table 4, the results showed that the lower the powder’s moisture content, the higher the hygroscopicity. This finding appears to be well supported by the results obtained by Goula et al. [48], on spray drying of tomato pulp. Meanwhile, Tonon et al. [11] suggested that the difference in moisture adsorption is due to the number of hydrophilic groups present in the structure of each wall material. They also reported that the high number of ramifications, with hydrophilic groups present in gum Arabic can easily absorb moisture from the ambient air compared to maltodextrin.

### 2.6. Dissolution Test

Dissolution test was conducted to measure the reconstitution speed of encapsulated cell powder into water. The fastest dissolution time can be observed on MFP and MFS samples compared to the two samples (Table 5). In general, dissolution or solubility depends mainly on the type of carrier agents used. In this study, the addition of miracle fruit plant parts to the gum Arabic dramatically helped to increase the dissolution time. Furthermore, powder size is another factor that may influence the dissolution rate. The smaller the size of powder, the larger the surface area of powder, thus increasing the dissolution time [49]. The particle size produced after spray drying process is largely influenced by the feed pumping rate and compressed air flow rate [50]. Meanwhile, an increase in spray dryer inlet air temperature may result in a larger particle size due to higher swelling caused by the higher temperature applied [51].

### 2.7. Survivability of L. lactis Gh1

#### 2.7.1. Survivability in Simulated Gastric Juice

**Figure 2:**
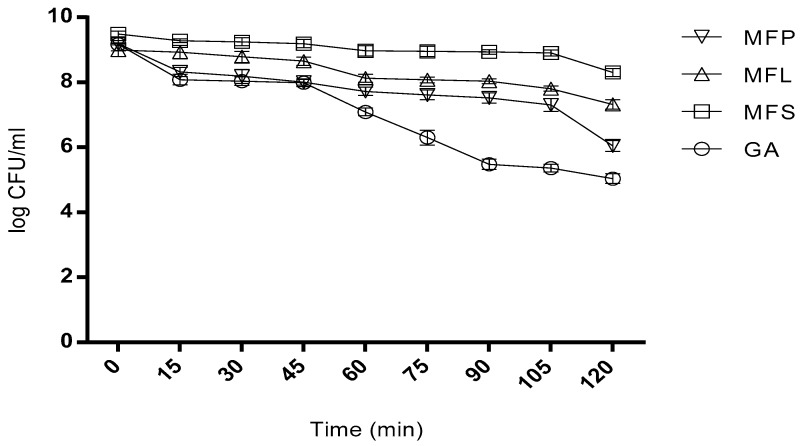
Viability of spray dried encapsulated *L. lactis* cells after exposure to simulated gastric juice at pH 1.55 (without pepsin) 37 °C for two hours. GA: Spray dried *L. lactis* Gh1 encapsulated with gum Arabic; MFS: Spray dried *L. lactis* Gh1 encapsulated with gum Arabic and miracle fruit seed; MFL: Spray dried *L. lactis* Gh1 encapsulated with gum Arabic and miracle fruit leaf; and MFP: Spray dried *L. lactis* Gh1 encapsulated with gum Arabic and miracle fruit pulp. The error bars represent the standard deviations about the mean (*n* = 3).

As depicted in Figure 2, even after 2 h of incubation, *L. lactis* cells viability can still be detected in all tested samples. In the first 15 min, the viability of spray dried *L. lactis* encapsulated in GA, MFS and MFL sharply decreased, meanwhile in MFP, the cell viability was observed to slowly decline at a more constant pattern. At 2 h, the cell viability in GA, MFS, MFL, and MFP were reduced to 4 log cycles, 1 log cycle, 2 log cycles, and 3 log cycles, respectively. The entire miracle fruit samples mixed with gum Arabic exhibited a much lower log reduction compared to GA sample. This observation highlights the roles played by miracle fruit parts to serve as a good encapsulant agents in supporting the gum Arabic. In summary, MFS sustained high cell viability of 2.0 × 10^8^ CFU/mL at 2 h while GA showed the lowest cell viability of 1.09 × 10^5^ CFU/mL.

#### 2.7.2. Survivability in Bile Salt

**Figure 3:**
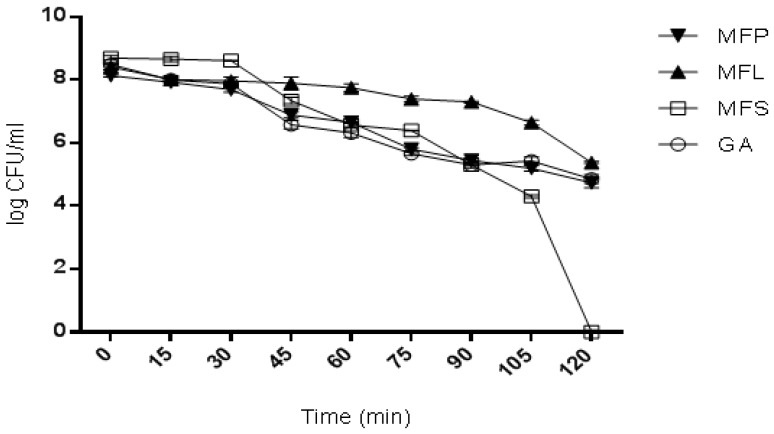
Viability of spray dried encapsulated *L. lactis* cells after exposure to simulated 0.6% bile salt of pH 8.25 at 37 °C for two hours. GA: Spray dried *L. lactis* Gh1 encapsulated with gum Arabic; MFS: Spray dried *L. lactis* Gh1 encapsulated with gum Arabic and miracle fruit seed; MFL: Spray dried *L. lactis* Gh1 encapsulated with gum Arabic and miracle fruit leaf; and MFP: Spray dried *L. lactis* Gh1 encapsulated with gum Arabic and miracle fruit pulp. The error bars represent the standard deviations about the mean (*n* = 3).

In general, a similar trend was observed on the cell survivability for all the cell samples when exposed to bile salt for 2 h (Figure 3). Even though a decline was noted for all samples, the cells were still survived after 2 h of exposure except for MFS. In sample MFS, the cells were surprisingly found to constantly decline in the first 30 min, and then sharply reduced until 120 min. Meanwhile, for the other three remaining samples, a constant decline without any major reduction was observed. At 1.45 h, the reduction of viable cells counted for GA, MFS, MFL, and MFP was approximately 3 log cycles. Nevertheless, no viable cell was detected at 2 h for MFS sample. This finding indicates that MFS had failed to sustain the cell viability in simulated bile salt solution at 2 h despite showing the highest survival rate in simulated gastric juice (Section 2.7.1). Overall, MFL showed the highest survival of cells after 2 h of incubation corresponding to 2.4 × 10^5^ CFU/mL.

### 2.8. Inclusion of Microencapsulated Probiotic L. lactis Gh1 into Yogurt

#### 2.8.1. pH Determination of Yogurt

**Figure 4:**
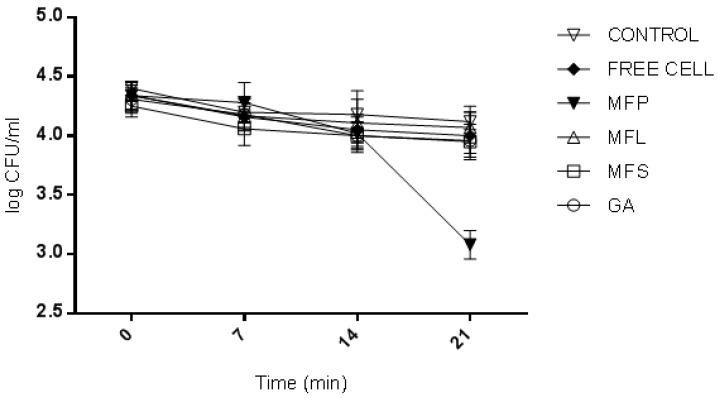
pH changes during 21 days storage at 4 °C of yogurts fortified with GA: spray dried *L. lactis* Gh1 encapsulated with gum Arabic; MFS: Spray dried *L. lactis* Gh1 encapsulated with gum Arabic and miracle fruit seed; MFL: Spray dried *L. lactis* Gh1 encapsulated with gum Arabic and miracle fruit leaf; MFP: Spray dried *L. lactis* Gh1 encapsulated with gum Arabic and miracle fruit pulp; Free Cells: *L. lactis* Gh1 free cell (non-encapsulated); and Control: Absent of *L. lactis* Gh1. The error bars represent the standard deviations about the mean (*n* = 3).

As can be seen in Figure 4, during the 21 days storage at 4 °C, a linear decline of pH can be observed in all yogurt samples, except for MFS, which sharply declined after day-14. At day-0, storage (determined after the yogurt fermentation had stopped), the highest pH was recorded in control yogurt (pH 4.4), while the lowest was MFS (pH 4.25). In other yogurt samples, the pH values at day-0 ranged from 4.31 to 4.34 with MFL, MFP and free cell having the same pH of 4.34 followed by GA, with a slightly lower pH of 4.31. In general, all the yogurt samples were observed to have a reduction in pH throughout the storage period. At day-21 of storage, the pH ranged from 3.08 to 4.12, with the highest value observed in control yogurt (absent of *L. lactis*) sample (pH 4.12), followed by MFL (pH 4.07), free cells (pH 4.0), GA (pH 3.96), MFS (pH 3.95), and the lowest was recorded for MFP (pH 3.08).

Generally, pH will be reduced in yogurt upon storage. During the fermentation process, lactic acid is produced by yogurt starter cultures, as they utilized the lactose component of milk. Typically, the process is known as acidification and gelation [52]. Furthermore, the addition of miracle fruit parts, as wall materials of the probiotic cell encapsulation may also contributed to the pH reduction. This claim can be supported by Senaka et al. [53], where 5%, 10%, and 15% concentrations of fruit juice had exhibited a lower pH compared to control yogurt produced by goat’s milk. Another report by Sah et al. [54], found that on the addition of pineapple peel powder into yogurt to act as a fiber, source attained a lower pH value, when compared to yogurt fortified with inulin as prebiotic.

The control yogurt exhibited a higher pH value than the other yogurts, which were mixed with encapsulated cells or non-encapsulated cells. Kailasapathy et al. [55], had concluded that the addition of fruits and other ingredients in yogurt will affect pH and cell viability. On the other hand, the addition of dietary powder fiber into yogurt reduces the fermentation time, as it provides additional carbohydrate sources for probiotics [54]. Therefore, the reduction of fermentation times observed for GA, MFS, MFL, and MFP yogurt samples were possible when compared to the control yogurt. Generally, reduction in fermentation time of commercial yogurt is preferable for reducing the manufacturing cost.

#### 2.8.2. Total Titrable Acidity (TTA) of Yogurt

**Figure 5:**
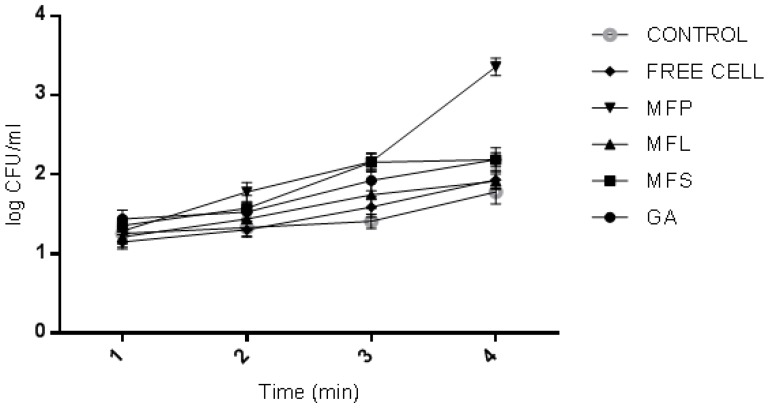
Total titratable acid (TTA) (% lactic acid) during 21 days storage at 4 °C of yogurts fortified with GA: spray dried *L. lactis* Gh1 encapsulated with gum Arabic; MFS: spray dried *L. lactis* Gh1 encapsulated with gum Arabic and miracle fruit seed; MFL: Spray dried *L. lactis* Gh1 encapsulated with gum Arabic and miracle fruit leaf; MFP: Spray dried *L. lactis* Gh1 encapsulated with gum Arabic and miracle fruit pulp; Free Cells: *L. lactis* Gh1 free cell (non-encapsulated); and Control: absent of *L. lactis* Gh1. The error bars represent the standard deviations about the mean (*n* = 3).

Figure 5 illustrated the total titratable acid (TTA) expressed in percentage of lactic acid, produced for all yogurt samples during the 21 day storage at 4 °C. At day-0 (determined after the yogurt fermentation had stopped), the highest TTA was recorded for GA (1.44%), followed by MFS (1.40%), MFP (1.29%), Control (1.26%), MFL (1.22%), and Free Cells (1.15%). A drastic increase in TTA was observed for MFP after 14 days of storage. The final TTA values, recorded at day-21, ranged between 1.78% to 2.19%. Overall, the highest TTA was measured for MFP, while the lowest was the control yogurt.

In this study, yogurt fortified with encapsulated *L. lactis* cells and free cells of *L. lactis*, showed higher values of TTA compared to the control yogurt (absent of *L. lactis*). These observations showed *L. lactis* strongly influenced the acidity behavior of yogurt. This finding is in agreement with do Espírito Santo et al. [56], who reported that the TTA in yogurt, supplemented with passion fruit peel powder, was higher than the control yogurt (absent of passion fruit peel powder). In addition, the type of probiotic strains being added to the yogurt also played an important role in the production of lactic acid. For instance, yogurt co-cultured with *Lactobacillus acidophilus* strains exhibited a lower TTA than *Bifidobacterium animalis*, due to the fact that homolactic metabolism of *L. acidophilus* produced 2 mol of lactic acid per glucose consumed, while *B. animalis* only produces 1 mol of lactic acid plus 1.5 mol of acetic acid [56].

The high TTA in MFP may be due to the addition of carbon sources for probiotics to produce lactic acid. It also can be deduced that the reduction of pH observed (Section 2.8.1) was caused by lactic acid production. In addition, the post-acidification of yogurt during refrigeration, may be due to the metabolic activity of bacteria. For instance, β-galactosidase produced by LAB was found to be active even at temperatures 0 °C to 5 °C [57]. In general, the acidity level in yogurt may also be influenced by the starter culture strains composition, storage duration, fermentation temperature, as well as contamination [58].

#### 2.8.3. Yogurt Morphology

**Figure 6:**
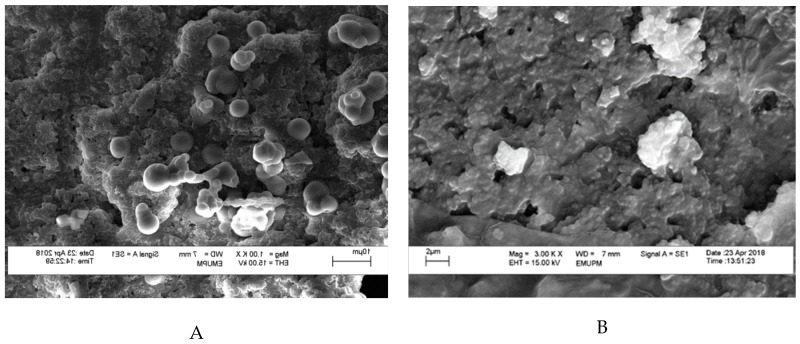
Scanning electron micrographs (SEM) of non-encapsulated and encapsulated *L. lactis* in yogurt during day 7 of storage at 4 °C. (**A**) spray dried *L. lactis* Gh1 with 5% gum Arabic (GA); and (**B**) non-encapsulated *L. lactis* Gh1 (Free Cells). Samples were magnified at 3,000×, and 10,000×, respectively.

Prior to viewing under scanning electron microscopy (SEM), the yogurt samples were freeze-dried to remove all the moisture present in the samples. All yogurt samples containing encapsulated cells were seen having a similar view under SEM without any obvious features to distinguish from one another. The freeze drying steps may have affected the hydrogen bonds involved in binding the surface layer of protein to the cell wall of bacteria in yogurt [59]. This may be the reason for the structural collapse in the morphology of all yogurt samples, and the presence of cells cannot really be distinguished. Hence, yogurt fortified with GA was chosen as a representative to compare with yogurt fortified with non-encapsulated *L. lactis* Gh1 (Free Cells). In Figure 6A, spherical encapsulated cells can be viewed adhering on the yogurt’s surface. In contrast, no encapsulated cell can be observed in Figure 6B. The variety of encapsulated cell sizes can be observed because of the freeze drying effect. Nevertheless, there is no large difference in size reduction that can be observed after freeze drying. Encapsulated cells had retained their shape and constituent during the 7 days of storage. This suggests that *L. lactis* was fully protected by the encapsulant agent (i.e., gum Arabic), thus increases cell survivability rate during storage at 4 °C. The SEM also showed the compactness of globular shapes in yogurts which were resulted from the casein micelles aggregation [54].

#### 2.8.4. Viability of *L. lactis* Gh1 and Yogurt Starter Culture Strains (*Lactobacillus delbrueckii* subs. *bulgaricus* and *Streptococcus thermophilus*)

**Figure 7:**
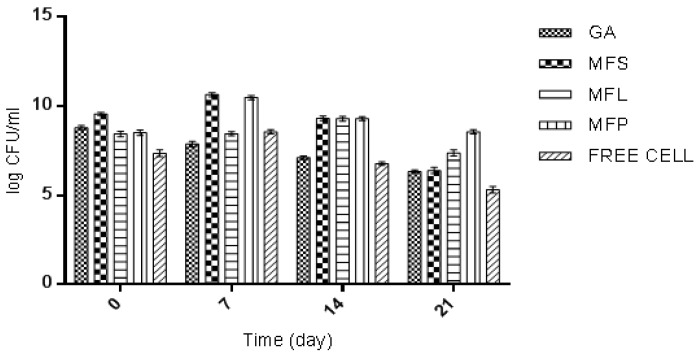
The viability of *L. lactis* Gh1 (log CFU/mL) in yogurts fortified with GA: Spray dried *L. lactis* Gh1 encapsulated with gum Arabic; MFS: Spray dried *L. lactis* Gh1 encapsulated with gum Arabic and miracle fruit seed; MFL: Spray dried *L. lactis* Gh1 encapsulated with gum Arabic and miracle fruit leaf; MFP: Spray dried *L. lactis* Gh1 encapsulated with gum Arabic and miracle fruit pulp; and Free Cells: *L. lactis* Gh-1 free cell (non-encapsulated) during 21 days of storage at 4 °C. The error bars represent the standard deviations about the mean (*n* = 3).

The efficiency of added LAB strains, in particular, *L. lactis* in yogurt formulation, depends on the initial dose level, cell viability maintained throughout the shelf life, as well as their survival in gut environment [60]. During day-0 of storage (determined after the yogurt fermentation was stopped), the yogurt sample fortified with MFS was observed to have the highest viability of *L. lactis*, which was 9.57 log CFU/mL, compared to other yogurt samples (Figure 7). Meanwhile the lowest *L. lactis* viability was 7.36 log CFU/mL as observed for yogurt with free cells. This result is as expected, given the fact that the free cells were not protected from the adverse external environmental conditions thus affecting their survivability. For the other yogurt samples, the *L. lactis* cell viabilities counted were in between 8.44 to 8.79 log CFU/mL according to the following order: GA > MFP > MFL. In general, *L. lactis* had exhibited an interesting trend. The viability of *L. lactis* increased from day-0 to day-7. Then it started to decline until day-21, except for GA, which decreased as the storage time increases. On 21 days of storage, the free cells yogurt showed the lowest cell viability (5.32 log CFU/mL) while the highest was observed in MFP yogurt (7.38 log CFU/mL). The order of cell viability was MFP > MFL > MFS > GA > free cells. This suggests that the present of miracle fruit pulp served as encapsulating agent that supporting the growth of *L. lactis* during storage.

Based on this study, the *L. lactis* encapsulated cells had survived better than the *L. lactis* of free cell form in yogurt. This observation can be supported by the work reported by Kailasapathy [61], where encapsulated *L. acidophilus* and *Bifidobacterium lactis* had increased the survival rate of 2, and 1 log CFU/mL, respectively. A similar result was also reported by do Espírito Santo et al. [56], where an increase in probiotic viable cells was observed when yogurt was fortified with acai pulp. MFS and MFP had shown an increase in the survival rate of 1 log, and 2 log CFU/mL, respectively. In general, the international dairy federation recommends that food products at the time of consumption should contain at least 10^6^ CFU/mL of probiotics to certify the beneficial effects [62]. All yogurt samples in this study had met the requirement at day 21 of storage, except for free cells yogurt. This highlights the significant of adding probiotic in an encapsulated form.

**Figure 8:**
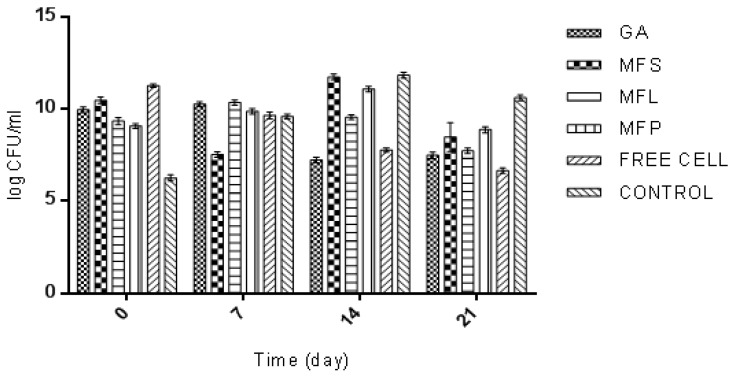
The viability of *L. delbrueckii* (log CFU/mL) in yogurts fortified with GA: Spray dried *L. lactis* Gh1 encapsulated with gum Arabic; MFS: Spray dried *L. lactis* Gh1 encapsulated with gum Arabic and miracle fruit seed; MFL: Spray dried *L. lactis* Gh1 encapsulated with gum Arabic and miracle fruit leaf; MFP: Spray dried *L. lactis* Gh1 encapsulated with gum Arabic and miracle fruit pulp; Free Cells: *L. lactis* Gh-1 free cell (non-encapsulated); and Control: absent of *L. lactis* Gh1 during 21 days of storage at 4 °C. The error bars represent the standard deviations about the mean (*n* = 3).

Meanwhile, Figure 8 illustrates the viability of yogurt starter culture strain, *L. delbrueckii* in six different yogurt samples. During day-0 of storage (determined after the yogurt fermentation was stopped), free cells yogurt (11.26 log CFU/mL) was found to have the highest *L. delbrueckii* viability, followed by MFS (10.47 log CFU/mL), GA (9.96 log CFU/mL), MFL (9.34 log CFU/mL), MFP (9.08 log CFU/mL, and Control (6.26 log CFU/mL). Cell viability trends during the 21 days of storage fluctuated, but it can be understood that, eventually, there was a decline in the viability of *L. deulbeuckii*. During 21 days of storage, the control yogurt (10.6 log CFU/mL) was observed to have the highest *L. delbrueckii* cell viability, while the lowest cell viability was measured for free cells yogurt (6.65 log CFU/mL). The order of *L. deulbeuckii*’s viability was Control > MFP > MFS > MFL > GA > Free Cells. This finding suggested that the addition of encapsulated *L. lactis* into yogurt had reduced the viability of *L. deulbeurkii* during the 21 days of storage.

**Figure 9:**
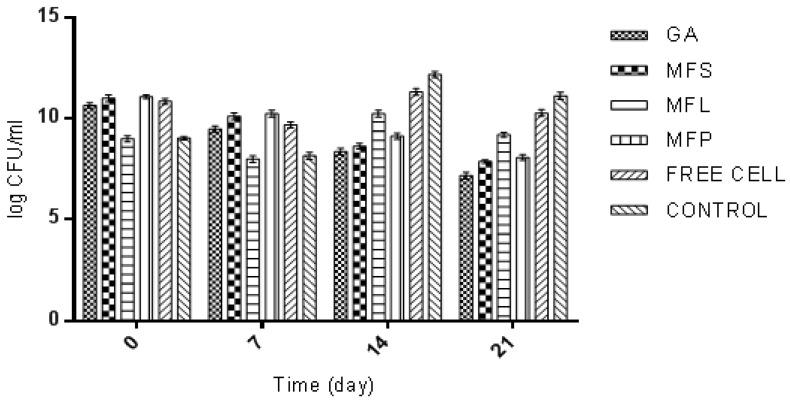
The viability of *S. thermophilus* (log CFU/mL) in yogurts fortified with GA: spray dried *L. lactis* Gh1 encapsulated with gum Arabic; MFS: Spray dried *L. lactis* Gh1 encapsulated with gum Arabic and miracle fruit seed; MFL: Spray dried *L. lactis* Gh1 encapsulated with gum Arabic and miracle fruit leaf; MFP: Spray dried *L. lactis* Gh1 encapsulated with gum Arabic and miracle fruit pulp; Free Cells: *L. lactis* Gh-1 free cell (non-encapsulated); and Control: Absent of *L. lactis* Gh1during 21 days of storage at 4 °C. The error bars represent the standard deviations about the mean (*n* = 3).

The viability of another yogurt starter culture, *S. thermophiles* during 21 days of storage, was shown in Figure 9. At day-0 of storage (determined after the yogurt fermentation was stopped), the highest viable cell of *S. thermophiles* was recorded in MFP (11.08 log CFU/mL). In the meantime, the lowest viable cell was observed in MFL (9.0 log CFU/mL). For the remaining yogurt samples, the viable cells were ranged from 9.03 log to 11.0 log CFU/mL according to the following order: MFS > Free Cells > GA > Control. Nevertheless, at day-21, Control yogurt was observed to have the highest cell viability of *S. thermophilus* (11.12 log CFU/mL) while the lowest was GA (7.18 log CFU/mL).

Based on the results obtained, the yogurt which were co-cultured with encapsulated and non-encapsulated probiotic cells *L. lactis* had affected the viable cell count of both yogurt starter culture strains *L. delbrueckii* and *S. thermophilus*. In control yogurt, *L. delbrueckii* was observed to have the lowest cell viability at day-0, while at day-21 the bacterium showed the highest viability compared to the rest of yogurt samples. The same trend can also be observed for *S. thermophilus* cells viability. Senaka et al., [53] suggested that mixed cultures of probiotic in yogurt will result in poor growth as they compete for nutrients availability. However, this finding is contradicted with Donkor et al. [63]. They had observed an increasing viability of starter cultures in probiotics mixture. Hence it can be deduced that the type of probiotic strains present in the sample also affects the viability of yogurt starter cultures.

## 3. Materials and Methods

### 3.1. Milk, Lactococcus lactis Gh1, and Yogurt Starter Culture

Fresh and pasteurized cow milk (brand: Farm Fresh) was purchased from a local market. *Lactococcus lactis* Gh1, used in this study, was obtained from the culture collection of Bioprocessing and Biomanufacturing Research Centre, Universiti Putra Malaysia (UPM), Selangor, Malaysia. This lactic acid bacterium was isolated from Iranian traditional flavor enhancer prepared from milk by-product [28,29]. The starter culture was purchased from Chris Hansen (Denmark) as freeze-dried, containing a mixture of bacteria strains (YC 380): *Streptococcus thermophilus* and *Lactobacillus delbrueckii subs. Bulgaricus.*

### 3.2. Miracle Fruit Pulp, Seed and Leaf Preparation

Miracle fruit (*Synsepalum dulcificum*) was obtained from a local farm located in Selangor, Malaysia. Following the method described by Hernández-Carranza et al. [64], with a slight modification, the miracle fruit pulp and seed were lyophilized using a freeze dyer for 48 h. The miracle fruits’ leaves were dried in a hot air dryer (FDD-720, Protech, Malaysia) at 50 °C for 48 h until dry. Then, the dried pulp, seed, and leaf were blended using a blender and stored in an air-tight container at 4 °C until usage. Upon usage, 5% (*w*/*v*) of the samples were mixed with distilled water using a stirring hotplate at 60 °C for 15 min. The juice was then filtered using a muslin cloth to remove any remaining particles with size of more than 0.05 mm.

### 3.3. Cell Suspension Preparation

A single colony of *L. lactis* Gh1 was inoculated into 50 mL sterilized MRS broth and incubated at 30 °C for 18 h. Later, 5% (*v*/*v*) of the culture was transferred into 95 mL MRS broth and incubated at 30 °C for 16 h. Cell suspension was harvested at an early stationary phase where cell growth reaches maximum (~1.61 × 10^10^ CFU/mL) by centrifuging (5804 R, Eppendorf, Germany) at 10,000 rpm for 15 min at 4 °C. Finally, the cell pallets were obtained and washed with 0.1% (*w*/*v*) sterilized buffered peptone water.

### 3.4. Microencapsulation of L. lactis Gh1 via Spray Drying

#### 3.4.1. Sample Mixture Preparation for Spray Drying

The sample was prepared according to the protocols described by Ananta et al. [46], with some modifications. Using a magnetic stirrer, 5% (*w*/*v*) of each sample (pulp, seed, leaf) containing *L. lactis* pallets were mixed with 5% (*w*/*v*) of gum Arabic (ratio 1:1). The initial bacterial count of the mixture should contain at least 10⁹ CFU/mL before undergoing the spray drying process.

#### 3.4.2. Spray Drying Technique

The spray drying process was executed using a laboratory lab scale spray dryer (SD-06, LabPlant, Essex, UK). The inlet and outlet temperature were set at 130 °C, and 60 °C, respectively. The pump speed was set at 50 rpm, liquid flow rate at 35.25 mL/min controller setting at 50, air speed at 4.3 m/s and deblocker at fast mode. The sample mixture was agitated using a magnetic stirrer at room temperature during the whole process. The dried powder sample was collected at the cyclone base using a Schott glass bottle and stored at 4 °C prior to further analysis.

#### 3.4.3. Enumeration of Bacteria After Spray Drying

The modified method used in this study was based on Chaikham et al., [65]. Briefly, 1 g of spray dried powder sample was mixed well with 9 mL of 0.1 % (*w*/*v*) peptone solution using vortex. Then, a serial dilution was carried out. Finally, the samples were plated on MRS agar plate using a track plate method and was incubated at 30 °C for 48 h [66]. The viability of cells was then calculated. The encapsulation efficiency (EE) can also be calculated by using Equation (1).

Encapsulati on efficiency (EE) = (Log_10_*N*/Log_10_*N*_0_) × 100 (1)

where; *N* is the number of entrapped viable cells and *N*_0_ displays the free viable cells before encapsulation.

### 3.5. Characterization of the Microencapsuled L. lactis Gh1

#### 3.5.1. Morphology

The morphological examination was performed by Scanning Electron Microscope (SEM) (Leo 435 VP, Leo Electronic Systems, Cambridge, UK), where its images were systematically observed at 15 kV under a vacuum of 9.75 × 10^−5^ Torr. The samples used must be in a dried form prior to viewing under SEM. Thus, all yogurt samples were placed in −20 °C for 2 days before freeze-dried by Martin Christ Epsilon 1–80 freeze dryer to further reduce the moisture content. The freeze-dried samples were crushed and carefully mounted on a double stick carbon tape placed on aluminum stub. The samples were introduced into the chamber of the sputter coater and coated with a very thin film of metal gold/palladium (40–60 nm) and observed under SEM. The cells were viewed at 1,000×, 3,000× and 5,000× magnifications.

#### 3.5.2. Moisture Content

The moisture content was measured by using moisture content analyzer (A&D Weighing MX-50 Moisture Balance, Thetford, UK).

#### 3.5.3. Water Activity (A_w_)

By following method described by Fritzen-Freire et al. [67], the dried powder samples were stabilized at 25 °C for 30 min. Then, the water activity of sample was measured using water activity meter (AquaLab, Pullman, WA, USA).

#### 3.5.4. Hygroscopicity

One gram of sample was weighed in an airtight glass bottle. Then, the bottle was placed in a desiccator with saturated NaCl solution, providing a relative humidity of 75.3%, at 25 °C. After 7 days, the sample was weighed. The moisture gained was expressed as g of adsorbed moisture per 100 g of dry powder [68].

#### 3.5.5. Dissolution

According to the method described by Quek et al. [69], 0.5 g of powder was mixed with 1 mL of distilled water in 2 mL Eppendorf tube and vortex at half speed. The time for powder to completely dissolve was recorded.

#### 3.5.6. Survivability of Spray Dried Microencapsulated Cells in Bile Salt

The method proposed by Halim et al. [5], was used to determine the survivability of the sample in bile salt. Briefly, 1 g of dried powder sample was placed in a 15 mL Falcon tube, containing 10 mL of 0.6% (*v*/*v*) sterilized bile salt solution with pH 8.25. Subsequently, the tube was incubated at 37 °C. Then, the sample was taken at 15 min intervals up to two hours. Finally, the sample was washed with 0.1% peptone solution and the viable cell count was determined.

#### 3.5.7. Survivability of Spray Dried Microencapsulated Cells in Simulated Gastric Juice

The method used was previously reported by Rao et al. [70]. Briefly, 1 g of dried powder sample was placed in 15 mL Falcon tube containing 10 mL of sterilized stimulated gastric juice (0.08 M HCL with 0.2% (*v*/*v*) NaCl without pepsin) at pH 1.55. Later, the tube was incubated at 37 °C and sample was taken at 15 min interval up to 1 h. Finally, the viability of cells was determined.

### 3.6. Yogurt Starter Culture Preparation

Yogurt starter culture was prepared following the method described by Amirdivani [71], with some modifications. Briefly, the freeze-dried yogurt bacteria were inoculated in fresh pasteurized cow milk (Farm Fresh, Johor, Malaysia) in a ratio of 1:100 (*wv*^−1^). The mixture was then incubated at 42 °C overnight without shaking. The yogurt formed was keep at 4 °C and used as a starter culture within 1 week.

### 3.7. Preparation of Yogurt

The yogurt was prepared following the method described by Shori and Baba [72], with some modifications. Six types of yogurt were prepared in this study (yogurts fortified with (i) GA: Spray dried *L. lactis* Gh1, encapsulated with gum Arabic; (ii) MFS: Spray dried *L. lactis* Gh1 encapsulated with gum Arabic and miracle fruit seed; (iii) MFL: Spray dried *L. lactis* Gh1, encapsulated with gum Arabic and miracle fruit leaf; (iv) MFP: Spray dried *L. lactis* Gh1 encapsulated with gum Arabic and miracle fruit pulp; (v) Free Cells: *L. lactis* Gh1 free cell (non-encapsulated); and (vi) Control: Absent of *L. lactis* Gh1). Briefly, 10 g of yogurt starter culture was added into 90 mL of fresh milk. For the yogurt supplemented with the encapsulated *L. lactis*, 3% (*w*/*v*) spray dried encapsulated cell were added, whereas for yogurt supplemented with non-*encapsulated L. lactis* 3% (*v*/*v*), free cell was added [73]. Then, the milk was incubated at 42 °C and pHs of the yogurts were taken every 30 min until it reached 4.5. Finally, the incubation of yogurts was stopped by placing it in a chiller (4 °C) for up to 21 days.

### 3.8. Analysis of Yogurt Sample

#### 3.8.1. pH and Total Titrable Acid (TTA) Determination

Yogurt samples were homogenized in distilled water with a ratio of 1:1 for pH measurement and 1:9 for TTA determination [61]. The pH value was measured using a pH meter (HI-2211, Hanna Instruments, Bedfordshire, UK). For TTA, the yogurt samples were added with 5 drops of 1% (*w*/*v*) phenolphthalein and titrated with 0.1 M of NaOH, until a pink color was obtained. The volume of NaOH was recorded and the percentage of lactic acid was calculated (Equation (2)).

Percentage of lactic acid (%) = d f (10) × V NaOH × 0.1N × 0.009 × 100%(2)

where,

V = Volume of NaOH required to neutralize the yogurt samples

N = Normality of the sodium hydroxide

Df = Dilution factor

#### 3.8.2. Microbial Viable Cell Count in Yogurts

##### Sample Preparation

YOGURT samples (0.1 g) were mixed with 0.9 mL sterile buffered peptone water. The mixture was mixed by vortex and a serial dilution was prepared by using buffered peptone water.

##### Enumeration of *S. thermophilus* Using Track Plate Technique

*S. thermophilus* was enumerated using M17 agar, as described by Shori and Baba [72], with some modifications. The M17 agar was prepared following the description given, followed by autoclaving at 121 °C for 15 min. The melted agar was allowed to cool (45 °C) before addition of sterilized 10% (*w*/*v*) lactose solution. The mixture was then placed in a petri dish and allowed to set. The agar plate was divided into 4 parts and 10 μL of diluted yogurt sample was transferred on each division of the agar surface. The plate was then held vertically to allow the sample to flow through the agar surface. The plates were sealed with parafilm and incubated at 37 °C for 48 h in inverted position. Viable *S. thermophilus* count was calculated by using colony forming unit (CFU) [74] as follows (Equation (3)):

(3) $$\frac{CFU}{mL}=\frac{Number\;of\;colonies\;formed\;\times dilution\;factor\;of\;sample}{1\;mL\;of\;sample}$$

where, CFU is colony forming unit.

##### Enumeration of *Lactobacillus delbrueckii* subs. *bulgaricus* Using Pour Plate Technique

*L. delbrueckii* was enumerated using MRS agar, as described by Shori and Baba [72]. The MRS agar was prepared following the description given, followed by autoclaving at 121 °C for 15 min. The melted agar was allowed to cool (45 °C) before poured in a petri dish. An aliquot of 1 mL of diluted yogurt sample was then transferred in the molten agar. The mixture was swirled slowly and allowed to set before the plates were sealed with parafilm and incubated at 37 °C for 48 h in an inverted position. Viable *Lactobacillus* sp. count was calculated following Equation (3).

##### Enumeration of *L. lactis* Gh1 Using Track Plate Method

The *L. lactis* was enumerated on MRS agar using track plate method as described in Section Enumeration of *S. thermophilus* Using Track Plate Technique. Then, the plate was incubated at 30 °C for 48 h. The viability of the cells was then calculated.

### 3.9. Statistical Analysis

All experiments were performed on at least three different occasions (*n* = 3). All data is expressed as a mean value ± standard deviation. Data analyses was performed on both Microsoft excel (2013) and Graph Pad prism 6 (GraphPad Software Inc., San Diego, CA, USA). The graphs for the data were plotted on Graph Pad prism 6 and statistical significance was determined at *p* value < 0.05. Statistical analysis was done using the Analysis of variance (ANOVA) (GraphPad Software Inc., San Diego, CA, USA); *p* < 0.05 was considered significant.

## 4. Conclusions

The results of the present study suggested that microencapsulation via spray drying has been proven to be one of the most effective techniques for conserving viability and stability of potential probiotic strains. The incorporation of miracle fruit pulp in a mixture of gum Arabic, for encapsulating the *L. lactis* Gh1 during spray drying, contributed to better functionality, viability, as well as morphology. The supplementation of miracle fruit not only protects the bacteria cells during food processing and harsh gastric conditions but also have their own health beneficial effects when incorporated into food product. Further research can be carried out to discover the capability of the miracle fruit to act as a prebiotic agent. The addition of *L. lactis* and miracle fruit pulp into food products other than yogurt is also interesting to explore.

## Figures and Tables

**Table 1:** Survival percentages of *L. lactis* Gh1 cells in different samples upon spray drying.

Samples	CFU/mL before Spray Drying	CFU/mL after Spray Drying	Percentage of Survival (%)	Encapsulation Efficiency (%)
**GA**	4.5 × 10^9^ ± (0.02) ^ax^	1.52 × 10^9^ ± (0.03) ^dy^	33.78	94.09
**MFS**	4.0 × 10^9^ ± (0.05) ^dx^	3.4 × 10^9^ ± (0.07) ^ax^	85.00	99.27
**MFL**	4.3 × 10^9^ ± (0.09) ^cx^	1.55 × 10^9^ ± (0.31) ^cy^	36.04	95.43
**MFP**	4.4 × 10^9^ ± (0.11) ^bx^	1.6 × 10^9^ ± (0.21) ^by^	36.36	95.44

The standard deviation represents the standard deviation about the mean (*n* = 3). ^a,b,c,d^ Means values in the same column expressed with different superscript letters are significantly different at (*p* < 0.05). ^x,y^ Means values in the same row expressed with different superscript letters are significantly different at (*p* < 0.05).

**Table 2:** Moisture content of spray dried encapsulated *L. lactis* Gh1.

Sample	Moisture Content (%)
**GA**	5.80 (±0.0707) ^a^
**MFP**	3.73 (±0.1980) ^c^
**MFL**	3.97 (±0.0424) ^b^
**MFS**	3.55 (±0.1273) ^c^

The standard deviation represents the standard deviation about the mean (*n* = 3). ^a,b,c^ Means values in the same column expressed with different superscript letters are significantly different at (*p* < 0.05).

**Table 3:** Water activity, *a_w_* of spray dried encapsulated *L. lactis* Gh 1.

Sample	Water Activity, *a_w_*
**GA**	0.3070 (±0.0014) ^a^
**MFP**	0.2925 (±0.0057) ^b^
**MFL**	0.2665 (±0.0049) ^c^
**MFS**	0.2620 (±0.0007) ^c^

The standard deviation represents the standard deviation about the mean (*n* = 3). ^a,b,c^ Means values in the same column expressed with different superscript letters are significantly different at (*p* < 0.05).

**Table 4:** Hygroscopicity (g/100 g of adsorbed moisture) of spray dried encapsulated *L. lactis*.

Sample	Hygroscopicity (g/100 g)
**GA**	0.0951 (±0.0012) ^c^
**MFP**	0.1092 (±0.0011) ^b^
**MFL**	0.1598 (±0.0005) ^a^
**MFS**	0.0952 (±0.0011) ^c^

The standard deviation represents the standard deviation about the mean (*n* = 3). ^a,b,c^ Means values in the same column expressed with different superscript letters are significantly different at (*p* < 0.05).

**Table 5:** Dissolution of encapsulated spray dried *L. lactis* Gh1.

Sample	Dissolution (minutes)
**GA**	2.12 (±0.0283) ^a^
**MFP**	0.38 (±0.0283) ^c^
**MFL**	0.54 (±0.0141) ^b^
**MFS**	0.39 (±0.0424) ^c^

The standard deviation represents the standard deviation about the mean (*n* = 3). ^a,b,c^ Means values in the same column expressed with different superscript letters are significantly different at (*p* < 0.05).
